# Risk and protective factors for GBV among women and girls living in humanitarian setting: systematic review protocol

**DOI:** 10.1186/s13643-021-01795-2

**Published:** 2021-08-28

**Authors:** Maureen Murphy, Mary Ellsberg, Aminat Balogun, Claudia Garcia-Moreno

**Affiliations:** 1 The Global Women’s Institute at the George Washington University, 2140 G Street NW, Washington DC, 20052 USA; 2 The Department of Sexual and Reproductive Health and Research, World Health Organization, Avenue Appia 20, 1211 Geneva 27, Switzerland

**Keywords:** Gender-based violence, Warfare and armed conflicts, Disasters, Risk, Protective factors, Refugees, Humanitarian

## Abstract

**Background:**

While one in three women around the world are estimated to have experienced intimate partner or sexual violence, these rates are often exacerbated during conflict and humanitarian crisis. This systematic review seeks to provide an overview of existing research on risk and protective factors associated with gender-based violence (GBV) in conflict and humanitarian settings.

**Methods:**

Studies will be searched from the following databases: PubMed (Medline); PsycINFO; Scopus; Global Health; and Cochrane Center trials registrar. In addition, targeted searches of the internet repositories for GBV will be conducted. We will include studies that are published between January 1995 and December 2020 and document risk or protective factors for gender-based violence against women and girls in conflict or humanitarian settings. Two reviewers will independently screen and extract data for the review, with a third reviewer arbitrating disputes and ensuring quality. A quality assessment of the included studies will be undertaken using a modified GRADE system. Narrative synthesis will be utilized to analyze the data.

**Discussion:**

The results of this study will inform the design and delivery of GBV prevention programs in conflict and humanitarian settings as well as contribute to the attainment of Sustainable Development Goal 5. The results will be published in a peer-reviewed journal and will be utilized at the World Health Organization to inform efforts to prevent GBV in conflict and humanitarian settings.

**Systematic review registration:**

The protocol has been registered with PROSEPERO (CRD42020198695).

**Supplementary Information:**

The online version contains supplementary material available at 10.1186/s13643-021-01795-2.

## Background

While one in three women around the world are estimated to have experienced intimate partner violence or non-partner sexual violence, two of the most common forms of gender-based violence (GBV), evidence shows that the risk of GBV is often higher in conflict and humanitarian crisis [1]. While rates of sexual violence vary by context, overall an estimated 21.4% of refugee and displaced persons in complex humanitarian emergencies have experienced such violence [2]. Even more women and girls experience intimate partner violence (IPV) compared to sexual violence alone and, in some conflict-affected contexts, rates of IPV as high as 73% of ever partnered women have been documented [3, 4]. Furthermore, exposure to armed conflict has been found to be associated with higher rates of IPV, suggesting that conflict and humanitarian crises directly and indirectly affect the drivers of multiple forms of GBV [4, 5].

There is still limited evidence on the scope and magnitude of non-partner sexual violence and IPV in conflict and humanitarian settings, and even less is known about other forms of GBV. For example, a systematic review of child, early and forced marriages in these settings found rates that ranged from 3 to 51% and no assessment was made about how armed conflict affected marriage rates [6]. The same study also examined rates of sexual exploitation and found there was not enough data to estimate prevalence. Similarly, other forms of GBV such as harmful practices (e.g., female genital mutilation, wife inheritance), trafficking, and femicide have not been consistently documented in conflict and humanitarian settings. Despite this, the limited research available has suggested that conflict and humanitarian settings can reinforce and potentially increase some of these practices. For example, adolescent girls in conflict settings may be married at early ages due to poverty or a desire to protect their virginity in contexts where conflict-related sexual violence is rampant [7].

No matter the context, there is agreement that the root cause of GBV is patriarchal gender norms and inequitable power dynamics and research has demonstrated the association between unequal gender norms and increased rates of IPV [8]. In conflict and humanitarian settings, programmers and academics believe that women and girls may be at heightened risk of violence for a variety of reasons including: displacement, the breakdown of social structures, a lack of law enforcement, the potential further entrenchment of harmful gender norms, and the loss of livelihood opportunities for both men and women in the community, among other reasons. In addition, active conflict dynamics (including the use of rape as a war tactic, breakdown of control of armed forces, increased availability of weapons, etc.) may also influence rates of GBV during these periods.

Since the 1995 United Nations Fourth World Conference on Women, there has been a considerable growth in research on GBV. Global initiatives such as the World Health Organization’s Multi-Country Study on Domestic Violence have standardized data collection measures and developed rigorous comparable evidence on prevalence and risk factors for GBV. In recent years, this increase in attention and rigor for GBV research has begun to expand into conflict and humanitarian crisis settings. Researchers have demonstrated it is possible to conduct rigorous prevalence studies [4] and impact evaluations [9–11] in these settings.

Despite the increasing evidence-base, gaps remain in our understanding of how conflict and other humanitarian crises impact rates of GBV. While researchers have conducted reviews of prevalence [2, 3, 6] and interventions [12–16], no systematic reviews have been conducted that specifically examine risk and protective factors that may influence GBV rates in these settings. This systematic review seeks to close this gap and provide an overview of existing research on risk and protective factors associated with GBV in conflict and other humanitarian settings. The findings of this review will inform practitioners and assist the field to develop more evidence-based prevention programming in conflict and humanitarian settings. In addition, the findings will be used to inform the World Health Organization to develop a GBV prevention framework for humanitarian settings.

## Methods/design

The findings of this systematic review will be reported in line with recommendations from the Preferred Reporting Items for Systematic Reviews and Meta-Analyses Protocols (PRISMA) statement [17].

### Review questions

The review questions that will be explored are the following:

1. What are risk factors for experiencing gender-based violence for women and girls living in humanitarian settings (conflict-affected, refugee/displacement, natural disaster)?
  - What are the risk factors for different age groups of women and girls?
  - What are the risk factors for women and girls with different vulnerabilities (e.g. disabled, single women, married adolescents)?

2. What are protective factors for experiencing gender-based violence for women and girls living in humanitarian settings (conflict-affected, refugee/displacement, natural disaster)?
  - What are the protective factors for different age groups of women and girls?
  - What are the protective factors for women and girls with different vulnerabilities (e.g., disabled, single women, married adolescents)?

### Objectives

To conduct a systematic review of quantitative and qualitative studies of risk and protective factors for GBV in conflict and humanitarian settings, in order to inform the design on GBV prevention programs in these settings.

### Study registration

The protocol has been registered with PROSEPERO (CRD42020198695).

### Patient and public involvement

No patient involved.

### Types of studies

This review will include research studies such as cross-sectional surveys, cohort and case-control studies, and qualitative studies. It will include data published in peer review articles as well as grey literature from non-governmental organizations (NGOs) or the United Nations. Existing systematic reviews will only be reviewed in order to identify potential original articles. Case studies will be excluded from the review.

### Types of participants and settings

This review will focus on the experiences of women and girls who reside in conflict and humanitarian settings. This includes locations affected by natural disasters, armed conflict, refugee or displaced populations, including resettled refugees, and displaced persons who are part of the European migrant crisis. It will include respondents who are currently living in these settings and retrospective studies where participants recall events that previously occurred while they were resident in a conflict or humanitarian settings. For resettled refugees, we will only include articles that detail GBV experienced while still living in a humanitarian crisis.

### Exposures

The review is examining the risk and protective factors for GBV. This could include known risk and protective factors identified in non-conflict-affected or humanitarian settings, such as drug and alcohol use, poverty, education, childhood experiences of violence, and could include risk or protective factors unique to conflict or humanitarian settings.

### Types of outcomes measures

The outcomes that will be the types of GBV experienced by women or girls. For this review, GBV will be defined as intimate partner violence (IPV—physical, sexual, psychological or economic), non-partner sexual violence, sexual abuse and exploitation, child, early and forced marriage, harmful practices (e.g., FGM), trafficking, abduction or femicide. These will be self-reported outcomes collected via household surveys, service-based data (e.g., health clinic records), or qualitative data.

### Information sources and search strategy

A reference librarian specializing in systematic reviews was consulted to develop the search terms and target databases. The search will cover literature published between January 1995 and December 2020 to cover a period that aligns with a considerable increase in rigorous research efforts in humanitarian settings and an increase in international attention on preventing and responding to GBV.

The following databases will be searched: PubMed (Medline); PsycINFO; Scopus; Global Health; and Cochrane Center trials registrar. In addition, targeted searches of the following internet repositories will be conducted: What Works to Prevent Violence against Women and Girls Evidence Hub (https://www.whatworks.co.za/); Prevention Collaborative Knowledge Platform (https://prevention-collaborative.org/knowledge-platform/); GBV Prevention Network (http://preventgbvafrica.org/understanding-vaw/vaw-resources/); UN Women’s Global Knowledge Platform to End Violence against Women (https://evaw.unwomen.org/).

The general search strategy is attached in Additional file 1 and will be modified in line with the specific search functionality of each database. Grey literature databases will be manually searched by two reviewers to identify any potential articles that meet the search criteria.

### Data collection and analysis

#### Eligibility criteria of the studies

The inclusion criteria for the review will be the following:

- Peer-reviewed articles or grey literature (e.g., Non-governmental agency (NGO) reports, United Nations reports) published in English
- Studies published between January 1995 and December 2020
- Studies documenting risk or protective factors for GBV (IPV, non-partner sexual violence, sexual abuse and exploitation, child, early and forced marriage, harmful practices (e.g., FGM), trafficking, and femicide, or abduction)
- Studies of the experiences of women and girls in conflict or other humanitarian settings
- Observational study or baseline analysis of a community-based evaluation
- Articles that include either primary or secondary data analysis

The exclusion criteria for the review will be:

- Studies published in languages other than English
- Studies published outside the search dates or where the full text is not available through the searched database
- Studies that do not examine risk or protective factors for GBV in conflict or humanitarian settings
- Studies that do not utilize primary or secondary data analysis or are case studies.

### Data management of the studies

The research team will utilize COVIDENCE (www.covidence.org) to manage the systematic review process. Results of the individual searches will be uploaded, and duplicates will be removed by the software. The inclusion and exclusion criteria for the review will be uploaded and two reviewers will be assigned roles through the COVIDENCE platform. All final citations of the included studies will be managed in RefWorks.

### Data selection of the studies

Two reviewers will independently screen all titles and abstracts of the initially imported studies to assess their eligibility. Three categories will be utilized at this initial step: yes, no and maybe. Studies with two yes or two maybe votes will automatically advance to the full text screening. Studies with conflicting assessments (yes/no, yes/maybe, no/maybe) will initially be discussed by the two reviewers to determine if agreement can be reached. For studies where agreement is not possible, a third reviewer will be the final arbitrator. The process will be repeated with those studies that advance to the full text screening, with the possible assessments being reduced to yes or no. Two reviewers will initially independently vote and come together to discuss disagreements. A third reviewer will make the final assessment in the event consensus is not achieved.

### Data extraction

Two reviewers will independently extract all relevant data items (e.g., risk and protective factors identified in the study, type of GBV) for the review. A third reviewer will randomly cross-check a selection of these to ensure no errors are made. Any disagreement between the two initial reviewers will also be resolved by this third reviewer. See Additional file 2 for a list of the data items that will be extracted. Data will be extracted and managed in Covidence.

### Data items

The full list of data items to be extracted can be found in Additional file 2. They include (1) general information and characteristics of the study, including the context of the study (e.g., armed conflict, natural disaster); (2) methodology, including the measures utilized, sample size and data analysis techniques; and (3) results, including identified protective and risk factors and the types of GBV explored.

### Data synthesis and analysis

Extracted data will be analyzed utilizing narrative synthesis. For this, data will be grouped under a framework organized by the type of violence under investigation. To consider heterogeneity between quantitative versus qualitative results, two separate tables—one focusing only on qualitative studies and one focusing on quantitative—will be created. This will allow us to identify commonalities and differences in the results between the two types of data collection methods. For each table and then the resulting narrative synthesis common risk and protective factors for each type of violence will be categorized. During this process the evidence in support of each risk or protective factor will be assessed (e.g., study design, quality, strength of association if available). In general, the review will rely on the results reported in the published studies; however, the authors may request additional information or clarification from the corresponding authors of the studies if needed.

### Appraisal/assessment of the quality of the included studies

After extraction, the study team will assess the quality of each individual study by examining the data collection methodology, sample size, data collection tools, and sampling methodology. Assessment of the quality of individual quantitative studies will be assessed using a criteria established by Rubenstein et al. for examining quantitative risk and protective factors for interpersonal violence [18]. This process includes a 7-point scale that includes (1) use of population-based sampling methods, (2) adequate sample size (500 participants), (3) adequate response rate (reported and 80%), (4) use of an established instrument for measuring violence, (5) clearly stated definitions for predictors, (6) study design accounts for temporality between predictors and violence, and (7) analysis captures different levels of violence or comparison process (e.g., linear regression, multinomial regression). This results in 0–7 point scale with 7 being highest quality.

For qualitative studies, quality will be assessed by adapting the criteria laid out in Mays and Pope [19]. To guide our quality assessment, a 5-point scale will be utilized considering (1) use of triangulation and perspectives of multiple stakeholder groups; (2) respondent validation; (3) clear exposition of methods of data collection and analysis; (4) reflexivity (sensitivity to the roles and biases of the data collectors and prior assumptions); and (5) attention to negative cases (details on contradictions or cases/opinions that deviate from the majority).

Finally, we will analyze the breath of the evidence included in the review using a modified GRADE system (https://www.bmj.com/content/336/7650/924) using the below categories:

- *High quality*—further research is very unlikely to change our confidence in the conclusion
- *Moderate quality*—further research is likely to have an important impact on our confidence in the conclusions
- *Low quality*—further research is very likely to have an important impact on our confidence in the conclusions
- *Very low quality*—any conclusion is very uncertain

Two reviewers will independently review and make the initial assessment. A third reviewer will arbitrate any disagreement and make the final assessment if needed.

### Presenting and reporting the results

Results of the review will be presented by type of GBV and then identified risk and protective factors. A PRIMA flowchart will be utilized to document the main steps and results of the review process itself. Data will be summarized narratively and with tables to summarize the key findings of each individual study. Quantitative and qualitative studies will be presented separately in tables and jointly considered in the narrative.

### Ethical issues

As all data in review will be extracted from previously published studies, the study does not meet the requirements of human subject’s research and as such has been exempted from Institutional Review Board (IRB) review.

### Publication plan

The review will be published in a peer-reviewed journal and will be utilized internally at the World Health Organization to inform efforts to prevent GBV in conflict and humanitarian settings.

## Discussion

GBV is a considerable challenge for women and girls in conflict and humanitarian settings. However, despite wide recognition of the scope of the problem, programs to prevent this violence and support survivors are often not evidence-based. The challenge of collecting rigorous data on GBV in these settings is immense, and there have been few academic studies exploring these issues due to limited funding, security issues, and other constraints. Given the difficulties in collecting primary data, the humanitarian community needs to better utilize the existing data that has been collected to gather lessons and inform programming. This review will add considerable knowledge to the evidence base as it will systematically identify, organize, and analyze the data that is available on risk and protective factors for GBV in these settings. This data is essential for programming seeking to design effective programs to reduce the risks of GBV in these settings and prevent new incidences of violence. In addition, the findings will help inform efforts to achieve the 2030 Sustainable Development Goals (SDGs) target 5.2 (“Eliminate all forms of violence against all women and girls in the public and private spheres, including trafficking and sexual and other types of exploitation”) by consolidating known data on drivers and risk factors for GBV in humanitarian settings [20]. It will provide an evidence-based framework for the development of new prevention programs that will help achieve this goal.

## Supplementary Information

**Additional file 1.** Search Strategy for the review.

**Additional file 2.** Items for data extraction.

## Data Availability

Data sharing is not applicable to this article as no datasets were generated or analyzed during the current study.

## Abbreviations

GBV: Gender-based violence
IRB: Institutional Review Board
PRISMA: Preferred Reporting Items for Systematic Reviews and Meta-Analyses Protocols
