# Supplementary material for: Risk and protective factors for GBV among women and girls living in humanitarian setting: systematic review protocol
Source: Syst Rev. 2021 Aug 28;10:238. doi: 10.1186/s13643-021-01795-2 (PMC8403411; doi:10.1186/s13643-021-01795-2)
Supplement: Supplementary file 1 — Additional file 1. Search Strategy for the review. [file 13643_2021_1795_MOESM1_ESM.pdf]

PubMed

("Sex Offenses"[Mesh] OR "Gender-Based Violence"[Mesh] OR "Rape"[Mesh] OR "sexual assault"[All fields] OR "survival sex"[All fields] OR "forced marriage"[All fields] OR "sexual abuse"[All fields] OR "sexual violence"[All fields] OR "Rape"[All fields] OR "Domestic Violence"[All fields] OR “Intimate Partner Violence” [All fields] OR "Physical Abuse" [All fields] OR "violence against women"[All fields] OR "violence against girls"[All fields] OR "sexual exploitation" [All fields] OR "child marriage" [All fields] OR "early marriage" [All fields] OR "wife batt*" [All fields] OR “abduction” [All fields] OR “family violence” [All fields] OR “honor killing” [All fields] OR “femicide” [All fields] OR “killing of women” [All fields] OR “sexual harassment” [All fields] OR “traditional practices” [All fields] OR “female genital” [All fields] OR “sexual slavery” [All fields] OR “transactional sex” [All fields] OR “prostitution” [All fields] OR “trafficking” [All fields] OR “wife inheritance” [All fields])

AND

("Warfare and Armed Conflicts"[Mesh] OR "War Exposure"[Mesh] OR "War"[All fields] OR "War Crimes"[All fields] OR "Disasters"[Mesh] OR "Disaster*"[All fields] OR "Genocide"[All fields]) AND ("Persons"[Mesh] OR "Refugees"[Mesh] OR "Migrant"[All fields] OR "asylum seeker*"[All fields] OR "Survivors"[Mesh] OR "Military Personnel"[Mesh] OR "Veterans"[All fields] OR "Relief work"[Mesh] OR "Womens Health"[Mesh] OR "Survivor"[All fields])

AND

("Risk"[Mesh] OR "Causality"[Mesh] OR "Protective factors"[Mesh] OR "Mitigat*"[All fields] OR "vulnerab*"[All fields] OR "risk"[all fields])

PsychInfo

("Gender-Based Violence" OR "Domestic Violence” OR "Intimate Partner Violence" OR "Physical Abuse" OR "Rape" OR "violence against women" OR "violence against girls" OR "sexual assault" OR "survival sex" OR "sexual abuse" OR "sexual violence" OR "sexual exploitation" OR "forced marriage" OR "child marriage" OR "early marriage" OR "wife batt*" OR “abduction” OR “family violence” OR “honor killing” OR “femicide” OR “killing of women” OR “sexual harassment” OR “traditional practices” OR “female genital” OR “sexual slavery” OR “transactional sex” OR “prostitution” OR “trafficking” OR “wife inheritance”)

AND

("Warfare and Armed Conflicts" OR "War Exposure" OR "War" OR "War Crimes" OR "Disasters" OR "Disaster*" OR "Conflict-affected" OR "Genocide" OR "Refugees" OR "Migrant" OR "Displaced Person*" OR "Displace*")

AND

("Risk" OR "Causality" OR "Protective factors" OR "Mitigat*" OR "vulnerab*" OR "risk" or "protective" or "drivers")

Scopus

TITLE-ABS-KEY ("Gender-Based Violence" OR "Domestic Violence" OR "Intimate Partner Violence" OR "Physical Abuse" OR "Rape" OR "violence against women" OR "violence against girls" OR "sexual assault" OR "survival sex" OR "sexual abuse" OR "sexual violence" OR "sexual exploitation" OR "forced marriage" OR "child marriage" OR "early marriage" OR "wife batt*" OR "abduction" OR "family violence" OR "honor killing" OR "femicide" OR "killing of women" OR "sexual harassment" OR "traditional practices" OR "female genital" OR "sexual slavery" OR "transactional sex" OR "prostitution" OR "trafficking" OR "wife inheritance") AND TITLE-ABS-KEY ("Warfare and Armed Conflicts" OR "War Exposure" OR "War" OR "War Crimes" OR "Disasters" OR "Disaster*" OR "Conflict-affected" OR "Genocide" OR "Refugees" OR "Migrant" OR "Displaced Person*" OR "Displace*") AND TITLE-ABS-KEY ("Risk" OR "Causality" OR "Protective factors" OR "Mitigat*" OR "vulnerab*" OR "risk" or "protective" or "drivers") NOT DBCOLL (medl)
