# Supplementary material for: Risk and protective factors for GBV among women and girls living in humanitarian setting: systematic review protocol
Source: Syst Rev. 2021 Aug 28;10:238. doi: 10.1186/s13643-021-01795-2 (PMC8403411; doi:10.1186/s13643-021-01795-2)
Supplement: Supplementary file 2 — Additional file 2. Items for data extraction. [file 13643_2021_1795_MOESM2_ESM.docx]

# Additional file 2 - Data Items for Extraction

| 1. **General Information and characteristics of the studies**    1. Author´s name    2. Journal    3. Year of publication    4. Country    5. Context (e.g. armed conflict, natural disaster, etc.)    6. Study population (e.g women, adolescents, etc.) |
| --- |
| Methodology  - 1. Study design   2. Sample/number of participants   3. Measures   4. Data analysis technique |
| Results  - 1. Type(s) of GBV examined   2. Risk factors noted   3. Protective factors note   4. Strength of association (e.g significance levels, Odds Ratios, Risk Ratio, etc.) |
| **4. Other relevant information** |
